# The Endocrine Adipose Organ: A System Playing a Central Role in COVID-19

**DOI:** 10.3390/cells11132109

**Published:** 2022-07-04

**Authors:** Francesca Cinti, Saverio Cinti

**Affiliations:** 1 UOS Centro Malattie Endocrine e Metaboliche, UOC Endocrinologia e Diabetologia, Dipartimento di Scienze Mediche e Chirurgiche, Fondazione Policlinico Universitario A. Gemelli IRCCS, 00168 Roma, Italy; cinti_francesca@hotmail.com; 2 Dipartimento di Medicina e Chirurgia Traslazionale, Università Cattolica del Sacro Cuore, 00168 Roma, Italy; 3 Center of Obesity, Department of Experimental and Clinical Medicine, Marche Polytechnic University, 60126 Ancona, Italy

**Keywords:** adipocyte, adipose organ, white adipose tissue, brown adipose tissue, mammary gland, transdifferentiation, obesity, T2 diabetes, COVID-19, precision medicine

## Abstract

In the last 30 years the adipose cell has been object of several studies, turning its reputation from an inert cell into the main character involved in the pathophysiology of multiple diseases, including the ongoing COVID-19 pandemic, which has changed the clinical scenario of the last two years. Composed by two types of tissue (white and brown), with opposite roles, the adipose organ is now classified as a real endocrine organ whose dysfunction is involved in different diseases, mainly obesity and type 2 diabetes. In this mini-review we aim to retrace the adipose organ history from physiology to physiopathology, to provide therapeutic perspectives for the prevention and treatment of its two main related diseases (obesity and type 2 diabetes) and to summarize the most recent discoveries linking adipose tissue to COVID-19.

## 1. The Physiological Role of the Adipose Organ

Human behavior necessarily tends to satisfy three levels of needs: the first is necessary for the survival of the individual and the other two for the survival of the species. The first involves the search for and the intake of food, the second the search for a partner to procreate and the third the maintenance of the offspring.

**Figure 1:**
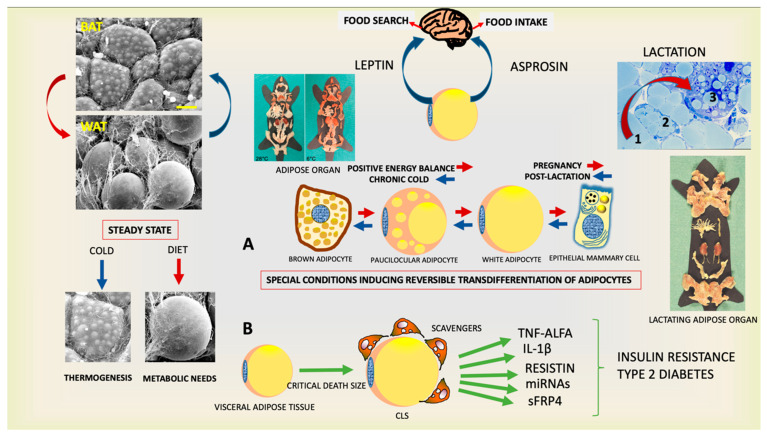
Plastic properties of the endocrine adipose organ. (**Upper left panels**): Scanning electron microscopy of brown (BAT) and white (WAT) adipose tissues contained in the adipose organ. (**Right upper panel**): Histology of adipo-epithelial conversion during pregnancy (transition steps are indicated by numbers). This endocrine organ (gross anatomy of mice shown at two temperatures) produces leptin and asprosin, hormones able to induce the primary behavior that allows the individual’s survival. Schematic representation of reversible transdifferentiation of adipocytes during special conditions such as cold exposure and pregnancy (**A**) and visceral adipose tissue involvement in the pathogenesis of insulin resistance and type 2 diabetes (**B**). Gross anatomy of adipose organ of mouse during lactation is also shown. Bar: 10 μm in BAT and 20 μm in WAT Modified from: Cinti S. Obesity, type2 Diabetes and The Adipose Organ, Springer 2018, with permission.

What plays the decisive role in inducing such behaviors? The hormones produced by the endocrine cells and the activation of the satisfaction center. The memory of how pleasant the outcome of behavior is, certainly plays an important role, but the action of hormones is crucial. For example, if we consider the primordial behavior of searching and eating food, we must reflect on the fact that we have understood the main underlining mechanisms only in the last 30 years. The group led by Jeffrey Friedman at Rockefeller University in New York City in 1994 discovered the hormone leptin, the absence of which leads to severe obesity [1]. The endocrine cell that produces this hormone was completely unexpected because it was considered almost inert for centuries and neglected by researchers: the adipocyte (Figure 1). In other words, the common fat cell. The anatomy of this cell suggests a poor functional activity because 90% of its volume is composed of a fat (triacylglycerols) vacuole surrounded by the vital elements of the cell (nucleus and cytoplasm) thin and squeezed at periphery by fat, suggesting their particular inertia and an energy reserve role. The term “reserve” in itself implies a secondary role, but the discovery of leptin has dramatically revived these cells not only elevating them to the endocrine role, but also defining them as determinants for primordial human behavior. The production of the hormone is proportional to the amount of fat, so when the energy reserves are thinned, leptin is reduced and this leads to the search for food [1,2,3]. Just a few years ago, a group led by Atul Chopra from Baylor College of Medicine at Houston added an important step. In fasting, the adipose cell produces another hormone called asprosin, which induces food intake [4,5]. The researchers came to this discovery by studying the gene responsible for severe congenital lipodystrophy: those affected are very thin essentially because they do not eat. Therefore, the low leptin levels of these subjects are not sufficient to stimulate the individual to consume food. These data show that the two hormones collaborate in inducing the primordial behavior for the survival of the human being: leptin stimulates the search for food while asprosin determines its intake. Another essential role of asprosin is to induce the liver to produce sugars, which are essential for the functioning of our brain. Other hormones intervene in food intake, but their role is secondary to that of the hormones produced and secreted by the adipose cell [6].

Thus, the adipocyte is far from being inert. Its spherical shape is dictated by the need to enclose the maximum amount (of energy) in the minimum space (only a sphere guarantees this advantage). This cell primarily supplies the body with essential energy molecules (lipids) for the functioning of the organ essential for survival: the heart, but also provides fuel for the functioning of the brain, which feeds mainly on sugars, with the help of asprosin and the liver.

After years of studies, it is now widely accepted that white adipose tissue can be considered an endocrine organ, able to secrete multiple peptide hormones, including leptin, asprosin, lipokines such as palmitoleato (C16:1n7), hundreds of cytokines known as adipokines [7] as well as exosomal miRNAs influencing the metabolism at different levels (i.e., glucose metabolism, lipid metabolism, reproduction, etc.) [8,9].

All organs have a complex cellular composition: at least two types of cells that collaborate for a common purpose and the adipose organ also contains another extremely interesting cell type: the brown adipocyte [10].

The term adipocyte is used because this cell also contains fat, but, unlike the adipose cell described above (white adipocyte), the fat in brown adipocyte does not form a single large vacuole, but a series of small vacuoles (Figure 1). So, these cells are smaller and the part of the organ that contains them is brown (hence the name) unlike the white color of the part of the organ (prevalent) that contains the common adipose cell called for this reason white adipocyte.

The anatomical differences always correspond to distinct functions and in fact those of the brown adipocyte could be defined as opposite to those of the white adipocyte: they burn fat to produce heat (thermogenesis) [11].

Thermogenesis is essential for survival because our body’s cells only function at 37 °C and most inhabited places are at lower temperatures. Beyond the thermogenesis role, the increase of brown adipocytes has been associated to multiple beneficial functions, such as anti-inflammatory effects, leading to an amelioration of glucose but also lipid metabolism [12]. Furthermore, the brown adipose tissue produces several hormone-like molecules (batokines), such as FGF21 and 12,13-diHOME, with cardioprotective properties [13].

Therefore, the fat cells are essential for the survival of the human being, but the excessive development of white adipose tissue is deleterious.

## 2. White Adipose Tissue: From Physiology to Disease

When the calories ingested exceed those consumed (positive energy balance) our body reacts by preserving what could be essential in the event of an upcoming unscheduled fasting period and develops the size of fat cells to become much larger (hypertrophy).

However, the expansion capacity has a limit that when reached triggers molecular mechanisms that eventually lead to the death of the cell [14,15,16].

A dead cell cannot remain inside the organism; the “scavengers” responsible for this purpose must remove its residues. The scavengers circulate in the blood ready to enter the organs where there are residues for elimination. They are inflammatory cells called macrophages (macros: large, phage: eats) because they eliminate the residues of dead cells and foreign bodies to the organism, but they are small in size compared to that of a dead hypertrophic adipose cell so they form particular structures called crown-like structures (CLS) in which the macrophages surround the cell residue (essentially the big lipid droplet) in a crown-like manner and work incessantly over a long period of time to permanently reabsorb the dead cell [16].

Their work is therefore commendable for the body, but the dead cells in the fat of the obese are many and, therefore, the inflammation becomes chronic. During this long process of cleaning the fat, the action of macrophages also determines a series of deleterious secondary effects. In fact, substances produced by macrophages interfere with the function of the insulin receptor [17,18]. Indeed, this pancreatic hormone, acting as a key, opens the “doors” of the cells and allows blood sugars to enter for their nourishment. If the door lock (insulin receptor) does not work, the insulin cannot let the sugars enter, resulting in high concentrations in the blood: that is, type 2 diabetes (T2D). This mechanism provides an explanation of the link between obesity and T2D (the most frequent, around 90%) [19,20] (Figure 1). In reality, this is achieved only after a period of compensation in which the insulin produced by the pancreas increases (hyperinsulinemia) to make up for its poor function (insulin resistance) and T2D develops when the exhausted insulin-producing beta cells are no longer able to compensate and undergo dedifferentiation in order to rest waiting for better metabolic conditions [21]. Recent data have demonstrated that noradrenergic fibers increase their density in pancreatic islets of obese mice and humans and this increased innervation is related to impaired insulin secretion and beta cell loss of identity (i.e., dedifferentiation) [22,23]. The reason why this happens is still object of research but, these new data, allow an easy interpretation of the very innovative discovery that bariatric surgery is able to revert T2D before weight loss [24]. Thus, a mechanistic interpretation is needed and warrants further studies.

So, obesity is an important cause of diabetes and explains why about 90% of diabetics are obese, but some types of obesity are more dangerous in causing diabetes. In fact, male obesity with increased abdominal fat, called apple-shaped obesity, is more predisposing to diabetes than female obesity with increased fat in the hips, called pear-shaped obesity [25]. Why?

The adipose cells that form abdominal fat (visceral) are less expansible than those of the hips (subcutaneous) and therefore when they enlarge, they die earlier causing more severe inflammation, which in turn induces diabetes [15,26].

Moreover, the set of adipose tissues forms a real (adipose) organ which in the lean comprises about 20% of body weight, but which in the large obese can reach 70% [27,28]. Therefore, it is a large organ that occupies both superficial (subcutaneous part) and deep districts near the viscera (visceral part) and its chronic inflammation can determine deleterious effects on the whole organism and there are increasing data correlating obesity to Alzheimer’s disease and some cancers [29,30,31,32].

## 3. COVID-19 and the Obese Adipose Organ

Adipose tissue has been proposed as a reservoir for SARS-CoV-2, since adipocytes express ACE2, the main receptor for this coronavirus [33]. Of note, patients with obesity overexpress several components for SARS-CoV-2 host cell entry (ACE2, CD147, DPP4 and NRP1), and spike protein processing enzyme (FURIN) in visceral fat, thereby increasing their susceptibility for SARS-CoV-2 infection [34]. Furthermore, proteomic and metabolomic studies have observed the upregulation of IFN-alpha pathways across all immune cell types with the increase of disease severity. They have also shown glucose itself and hyperinflammation are both associated with disease severity [35,36].

The COVID-19 disease, caused by SARS-CoV-2 infection, is characterized by bilateral pneumonia whose origin is still unclear [37,38].

Recent data suggest a peculiar increase of inflammation in the visceral fat of patients who passed away from COVID-19 [39]. In an autoptic observational study of biopsies from visceral fat, lungs and liver of 19 COVID-19 positive and 23 comparable (age and BMI) deceased patients from other causes, it was discovered that visceral fat was particularly inflamed and the number of macrophages (major responsible inflammatory element) doubled that found in controls with the same BMI and size of adipocytes. Electron microscopy revealed that adipocytes were frequently necrotic and free lipid droplets were often found in the interstitial space of visceral fat. Considering that in a previous work a hypothesis of lung fat embolism was proposed, based on the finding of several large lipid droplets in lung vessels and interstitial space and lipid-rich embolic material in mesenteric venous vessels of two patients who died from COVID-19 [40], the relationships between the free lipid droplets and vessels were studied. Several lipid droplets in endothelial cells, protruding into the lumen and free in the lumen were found. All data support the possibility that lipid remnants from dead adipocytes, probably caused by the hyper inflammation due to SARS-CoV-2 infection, invade vessels forming embolic material. In order to observe the consequences of viral infection on adipocytes, a well-established cell line of human adipocytes was used: hMADS [41]. hMADS infected by SARS-CoV-2, together with signs of reduced viability, showed clear aspects of delipidation further supporting the idea that infected fat extrudes free lipid droplets. A detailed study of venous vessels of visceral fat showed that about 11% of venous vessels of visceral fat were occupied by lipid-rich embolic structures. In line with these data, 100% of COVID-19 positive patients showed signs of lung fat embolism, suggesting that lipid droplets derived from necrotic adipocytes of visceral fat, give rise to fat embolism ending in the lungs. In order to reach the lung, embolic material must pass through the liver. In accordance, fat embolic material was found in sinusoids and blood vessels of liver biopsies in 8/9 COVID-19+ patients studied. Surprisingly, fat embolism, both in the lung and liver, was not exclusive of COVID-19 patients but significantly prevalent. Another surprising finding consisted in the discovery of the lipid nature of the hyaline membranes of COVID-19 patients (present in all COVID-19+ patients and only in one control patient). Hyaline membranes are a very important aspect of COVID-19 [42]. They consist in flattened structures arranged on the lung alveolar surface of COVID-19 patients, thus forming a barrier for the gaseous exchange of normal respiration and thus responsible for the reduced oxygen saturation of the blood in these patients. Transition aspects between roundish embolic material and progressively flattened lipidic material were also observed supporting the idea that hyaline membranes could derive for progressive flattening of embolic fat.

**Figure 2:**
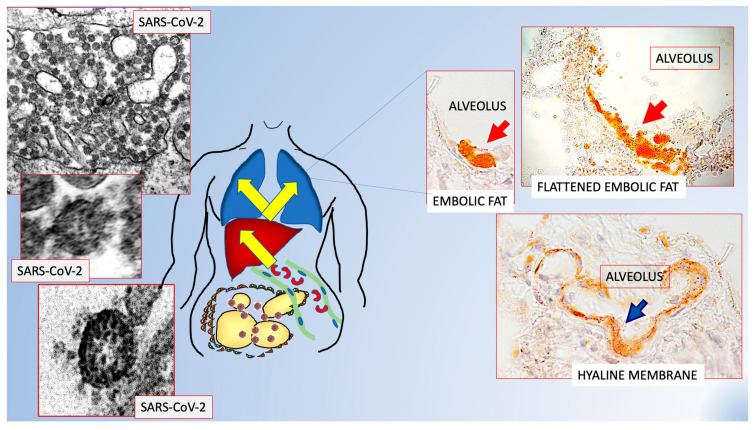
Scheme showing the possible role of fat embolism in COVID-19. Schematic representation of data from refs [39,40] that offer a possible explanation to the poorer prognosis for visceral obesity and sudden bilateral pneumonia in patients suffering from COVID-19. Visceral fat hyperinflammation could cause the fat embolism found in 100% of lungs of COVID-19+ patients. Original figures from Refs [39,40] with permission.

All these data offer an explanation to the fact that visceral fat abundance is a cause of poorer prognosis for COVID-19 patients [43,44,45,46] and for the clinical aspect related to the sudden bilateral pneumonia [37,38] (Figure 2).

Of note, these data are in line with another recent work showing an increased number of autoimmune antibodies against the malondialdehyde and the adipocyte-derived protein antigen (markers of lipid peroxidation and adipocyte death, respectively) among subjects with COVID-19 and obesity as compared to individuals suffering from each condition independently [47,48].

In another study, hyperglycemia among subjects with COVID-19 was found to be associated with insulin resistance and low plasma adiponectin levels. The authors also demonstrated that SARS-CoV-2 could infect hamster adipose tissue, leading to reduced adiponectin production and speculated that SARS-CoV-2 infection might result in adipocyte dysfunction inducing insulin resistance [49].

The presence of SARS-CoV-2 in the adipocytes of fat from COVID-19 patients has been demonstrated in a very recent paper that also showed chronic inflammation with upregulation of the interferon-alpha pathway [50]. Of note, interferon-alpha stimulates the expression of ACE2 and this type of condition has been reported in chronic lung diseases that appear to stimulate gene expression programs promoting both the cellular entry of SARS-CoV-2 and the severity of COVID-19 [51].

Type 2 diabetes is one of the four risk factors for long-COVID [36]. The cause of type 2 diabetes is quite complex but the chronic low-grade inflammation of obese adipose organ seems to play a relevant role (see above). In particular, TNFα, IL-6, resistin, galectin-3 and other molecules produced by macrophages interfere with insulin receptors causing insulin resistance [20]. Interestingly, the most injured organs involved in long-COVID (heart, intestine, skeletal muscles, kidneys) are endowed by visceral fat that is hyper inflamed in obese adipose organ of mice and humans, suggesting a connection between the two phenomena [10]. On the other hand, new onset of diabetes has been described in COVID-19 [52] and, recently, a direct effect of SARS-CoV-2 on human pancreatic islets has been proposed as a potential mechanism which may lead to metabolic dysfunctions [53].

In line with the above reported data, recent literature discovered metabolites that can predict patient survival in COVID-19 patients. Some of those metabolites, such as acetoacetate (produced in response to impaired glucose cellular uptake) and α-ketobutyrate (involved in the production of hydroxybutyrate34, an early insulin resistance marker) are related to glucose metabolism, insulin resistance and diabetes [36]. Insulin resistance can also be responsible for a lower uptake of glucose in immune cells, thus influencing their immunological activity, together with all the inflammatory consequences in fat causing fat embolism as described above.

Taken together, all these data underline the important role of adipose tissue in a systemic disease such as COVID-19, with relevant therapeutic implications which should be taken into account to change the prognosis of this infection.

## 4. What Prospects Are There?

As stated before, the adipose organ is composed by white and brown adipose tissue. Several studies have demonstrated that obesity in mice can be prevented and treated by stimulating brown adipocytes (fat burning) [54], and drugs capable of stimulating this component of the adipose organ could also have positive effects on human obesity and diabetes, leading to a precision medicine [55].

Some pharmaceutical companies are focusing their attention on the development of drugs [55,56] capable of activating brown adipocytes also in consideration of the fact that the stimuli capable of activating these cells are also able to stimulate a new and unexpected cellular property: transdifferentiation [57].

This phenomenon involves the direct transformation of one cell type into another (Figure 1). In fact, we have shown that, under physiological conditions, the white adipocyte can transform into brown adipocyte in both mice and humans, offering the possibility of exploiting the potential anti-obesity/diabetic capabilities of the latter cellular elements [57,58,59,60,61,62]. The physiologic stimuli able to induce white to brown transdifferentiation are cold exposure [63,64,65] and physical exercise [66,67]. Moreover, specific nutrients seem to have some influence in the white–brown transdifferentiation phenomenon [68,69]. Further, interesting data have paid attention to the role of mineralocorticoid receptor antagonism, providing a new potential browning inducer [70,71,72,73].

The enormous impact on the scientific community who have investigated this cellular conversion warrants the search for new examples. We have identified an even more striking one: the fat cells of the breast are transformed into glands for the production of milk during pregnancy and breastfeeding (Figure 1) and then return to form fat cells in post-breastfeeding [74,75,76,77,78,79].

Thus, these data suggest a new and exciting biological property of human cells: with appropriate physiological stimuli, they can reversibly change their identity and function. To trivialize, it would be as though a doctor had suddenly become an engineer, formed plans and then reverted back to a doctor and began treating patients.

Overall, it is clear that the reciprocal and physiologic white–brown adipose tissue transdifferentiation property could be safely used as therapy for obesity, type 2 diabetes and related disorders in the near future and recent pharmacologic approaches seem to be encouraging in this sense [55,56,80]. However, we should remember that physiologic stimuli such as cold exposure, physical exercise, and certain food could also help in this healthy phenomenon [64,66,67,69].

Furthermore, these discoveries provide hope that future studies can identify the fine molecular mechanisms able to change the identity and function of cells, perhaps even transforming neoplastic cells into normal cells.

## Data Availability

Not applicable.

## Abbreviations

T2D (type 2 diabetes), CLS (crown-like structures), brown (BAT) and white (WAT) adipose tissue, BMI (body mass index).
